# Behavioral Symptomatology in the Premenstruum

**DOI:** 10.3390/brainsci12070814

**Published:** 2022-06-22

**Authors:** Nhan Dang, Dina Khalil, Jiehuan Sun, Aamina Naveed, Fatimata Soumare, Robin Nusslock, Ajna Hamidovic

**Affiliations:** 1 Department of Public Health, University of Illinois at Chicago, Chicago, IL 60612, USA; ndhang@uic.edu (N.D.); jiehuan@uic.edu (J.S.); 2 Department of Public Health, Benedictine University, Lisle, IL 60532, USA; khalildina1@gmail.com; 3 Department of Pharmacy, University of Illinois at Chicago, Chicago, IL 60612, USA; anavee5@uic.edu (A.N.); fsouma2@uic.edu (F.S.); 4 Department of Psychology, Northwestern University, Evanston, IL 60208, USA; nusslock@northwestern.edu

**Keywords:** premenstrual syndrome, premenstrual dysphoric disorder, food cravings, sleep disorder, premenstrual eating/sleeping behaviors

## Abstract

Objective: Sleep and eating behaviors are disturbed during the premenstrual phase of the menstrual cycle in a significant number of reproductive-age women. Despite their impact on the development and control of chronic health conditions, these behaviors are poorly understood. In the present study, we sought to identify affective and psychological factors which associate with premenstrual changes in sleeping and eating behaviors and assess how they impact functionality. Methods: Fifty-seven women provided daily ratings of premenstrual symptomatology and functionality across two-three menstrual cycles (156 cycles total). For each participant and symptom, we subtracted the mean day +5 to +10 (“post-menstruum”) ratings from mean day −6 to −1 (“pre-menstruum”) ratings and divided this value by participant- and symptom-specific variance. We completed the statistical analysis using multivariate linear regression. Results: Low interest was associated with a premenstrual increase in insomnia (*p* ≤ 0.05) and appetite/eating (*p* ≤ 0.05). Furthermore, insomnia was associated with occupational (*p* ≤ 0.001), recreational (*p* ≤ 0.001), and relational (*p* ≤ 0.01) impairment. Conclusions: Results of the present analysis highlight the importance of apathy (i.e., low interest) on the expression of behavioral symptomatology, as well as premenstrual insomnia on impairment. These findings can inform treatment approaches, thereby improving care for patients suffering from premenstrual symptomatology linked to chronic disease conditions.

## 1. Introduction

Premenstrual syndrome (PMS) is one of the most commonly reported health problems in women of reproductive age. Affecting between 20 to 40% of menstruating women [1], it refers to a cluster of adverse symptoms, experienced in the late luteal phase, which resolve by the end of menstruation. The more severe form of PMS, premenstrual dysphoric disorder (PMDD), affects between 3–8% of reproductive-age women [1]. Changes in eating and sleep patterns in women affected by PMS and PMDD are particularly poorly understood, which is problematic as they can lead to the development and poor control of a multitude of chronic health conditions. 

Food craving, defined as “an intense desire to eat a specific food or food type” [2,3], has negative consequences on health due to its association with high body mass index [4] and eating disorders [5]. Women endorse increased food intake and food cravings in the premenstruum both retrospectively [6,7] and prospectively [8,9,10]. Indeed, functional magnetic resonance tomography studies show higher brain responses to food stimuli in the luteal vs. follicular phase, particularly in the corticolimbic areas involved in homeostasis and reward [11,12,13,14]. In addition to changes in food-related behaviors, women experience significant changes in sleep patterns in the premenstruum. According to the results of survey data, women with PMS frequently report sleep-related complaints, such as frequent awakenings and difficulty initiating sleep after arousal [15,16,17], which may have cardiometabolic consequences [18]. 

Despite the significance of eating and sleep disturbances on health, our understanding of affective and psychological factors which are associated with premenstrual changes in sleep and eating behaviors is limited as no study to date explored this topic. There is a theoretical basis for hypothesizing that affective and psychological symptoms may be related to eating and sleeping patterns in reproductive-age women. For example, ecological momentary assessment (EMA) studies have identified functional relationships between affect and various eating behaviors [19,20,21,22,23]; moreover, in the general population, insomnia is associated with apathy or low interest [24], as well as cognitive deficits [25].

In the present study, we used The Daily Record of Severity of Problems (DRSP) [26]—a validated questionnaire which measures premenstrual symptoms—to answer the following specific questions: (1) which affective (depressed mood, anxiety, mood swings, anger), and psychological (low interest, difficulty concentrating, feeling overwhelmed) symptoms associate with insomnia, hypersomnia, food cravings, and increased appetite/overeating in the premenstruum? (2) do premenstrual changes in sleep and eating (i.e., insomnia, hypersomnia, food cravings, increased appetite/overeating) associate with occupational, relational, and, recreational impairments? Our overarching goal was to improve the understanding of factors associated with changes in premenstrual eating/sleep behaviors, thereby contributing to improved preventive and therapeutic approaches for chronic diseases which associate with them. 

## 2. Materials and Methods

### 2.1. Study Design

The present analysis is a part of the Premenstrual Hormonal and Affective State Evaluation (PHASE) study in which reproductive-age women with regular menstrual cycles are enrolled to chart their symptoms using the Daily Record of Severity of Problems (DRSP) [26], menstruation timing and ovulation during three menstrual cycles. PHASE is described in detail in Hamidovic et al. [27]. PHASE is a registered clinicaltrials.gov study (NCT03862469).

### 2.2. Study Sample

The present study recruited reproductive-age women between the ages of 18 and 35 who self-reported regular menstrual cycles lasting 21 to 35 days [28]. 

Eligible participants were non illicit drug-using (urinary drug test-confirmed), non-smoking (carbon monoxide concentration < 5 ppm), and non-heavy drinking (Alcohol Use Disorders Identification Test (AUDIT) score < 8). They were not using any prescription medications, or hormonal birth control. Finally, the study participants did not have current (i.e., within the past 12 months) DSM-5 Major Depressive Disorder or an anxiety disorder (based on SCID). Detailed inclusion/exclusion criteria can be found in Hamidovic et al. [27]. 

Eight study participants met the diagnosis for PMDD, 25 met the diagnosis for PMS, and 24 participants were healthy. The PMDD group was oversampled in order to create a mix of participants with severe (PMDD), moderate (PMS), and no symptomatology (healthy). The diagnosis was made according to the Diagnostic and Statistical Manual-5 (DSM-5) criteria by evaluating prospective ratings of DRSP symptoms. The diagnosis for PMDD was defined as a 30% or greater increase in 5 or more symptoms, one of which had to be negative mood, relative to the range of the scale used by each woman during the week before compared with the week after menstruation [29,30]. In addition, every study participant with PMDD had to mark a 30% increase in functional impairment (occupational, relational, or recreational) during the week before vs. the week after menstruation. For PMS, a study participant had to have a 30% or greater increase in between 1 to 4 symptoms, without the requirement that one has to be affective, which is in accordance with DSM-5 criteria. 

### 2.3. Study Measures 

The Daily Record of Severity of Problems (DRSP) [26] is a questionnaire used for the assessment of premenstrual conditions (PMS and PMDD). The questionnaire contains 24 items (i.e., symptoms), which are rated on a scale between 1 (none) to 6 (extreme). The relationship between the 24 DRSP symptoms and the 11 PMS/PMDD domains is specified in Supplementary Table S1. In the present analysis, we evaluated affective, psychological, behavioral, and functional, symptoms of the questionnaire, leaving out physical symptoms, as they may be numerous and would require a larger sample size for a comprehensive evaluation of symptomatology. 

The following DSM-5 affective symptoms were evaluated (with specific DRSP questions in parentheses and including quotation marks): 1. Marked affective lability (e.g., mood swings, feeling suddenly sad or tearful, or increased sensitivity to rejection) (“Had mood swings (e.g., suddenly felt sad or tearful)”), 2. Marked irritability or anger or increased interpersonal conflicts (“Felt angry, irritable”), 3. Marked depressed mood, feelings of hopelessness, or self-deprecating thoughts (“Felt depressed, sad, down, or blue”), and 4. Marked anxiety, tension, and/or feelings of being keyed up or on edge (“Felt anxious, tense, keyed up, or on edge”). Psychological symptoms assessed were: 1. Decreased interest in usual activities (e.g., work, school, friends, hobbies) (“Had less interest in usual activities (e.g., work, school, friends, hobbies)”), 2. Subjective difficulty in concentration (“Had difficulty concentrating”), and 3. A sense of being overwhelmed or out of control (“Felt overwhelmed or that I could not cope”). The four behavioral symptoms from DSM-5/DRSP were: 1. Hypersomnia or insomnia (“Slept more, took naps, found it hard to get up when intended” and “Had trouble getting to sleep or staying asleep”), and 2. Marked change in appetite; overeating; or specific food cravings (“Had increased appetite or overate” and “Had cravings for specific foods”). Finally, we assessed functionality with the following DRSP questions: 1. Occupational impairment (“At work, school, home, or in daily routine, at least one of the problems noted above caused reduced productivity or inefficiency”), 2. Recreational impairment (“At least one of the problems noted above interfered with hobbies or social activities (e.g., avoided or did less)”), and 3. Relational impairment (“At least one of the problems noted above interfered with relationships with others”). 

### 2.4. Data Analysis 

For each participant and symptom, we calculated the degree to which the symptom demonstrated an elevation in days −6 to −1 (“pre-menstruum”) from the start of the cycle relative to days +5 to +10 (“post-menstruum”). We subtracted the average post-menstruum score from the average pre-menstruum score and divided this value by participant- and symptom-specific variance; this essentially yielded an effect size for each woman and for each symptom [8]. We used 12 days’ data of each cycle to calculate the effect size. The rate of missing data for all the participants across the 12 days was 10.3%, and the data were missing at random. Since effect sizes only need the average scores of the pre-menstruum and post-menstruum phases and participant-specific variances, we used the available data to calculate the average scores, variances, and effect sizes. We did not need to remove any participants due to missing data. The overall means and standard deviations for symptom ratings according to subphase can be found in Supplementary Table S2. 

We next evaluated which affective (depressed mood, anxiety, mood swings, anger) and psychological (low interest, difficulty concentrating, felt overwhelmed) symptoms associated with behavioral symptoms (insomnia, hypersomnia, increased appetite/eating and food cravings). We constructed four models (one model for each behavioral symptom) and entered effect sizes for the four affective and three psychological symptoms as predictors, with the effect size of a specific behavioral symptom as the outcome. These sets of models represented the unadjusted models. The adjusted models additionally included age and age of menarche, in order to account for the total time of reproductive hormone exposure based on the findings that onset of menarche [31] and age [32] are inversely associated with expression of premenstrual symptomatology. 

The final set of models evaluated which behavioral symptoms were associated with functionality. Three models were constructed for each impairment type (effect size for occupational, recreational, and relational) as outcomes, with effect sizes of the four behavioral symptoms as predictors. These models constituted unadjusted models; the adjusted models included age and age of menarche. 

We visually inspected and formally tested the distribution of residuals for each model using the Shapiro Test. We considered the results to be statistically significant at *p* ≤ 0.05, and marginally significant at *p* ≤ 0.1. All data analyses were conducted in R [33].

## 3. Results

### 3.1. Study Participants

Our study included 57 participants who were approximately 26 years old. Approximately 80% of the participants reported non-Hispanic ethnicity, and they were mostly white (33.3%) and Asian (36.8%). Please see Table 1 for additional participant characteristic details. 

Eight study participants met the diagnosis for PMDD, 25 met the diagnosis for PMS, and 24 participants were healthy. These groups did not differ according to demographic characteristics as specified in Supplementary Table S3.

### 3.2. Relationships between Behavioral, Affective and Psychological Premenstrual Symptoms

**Figure 1:**
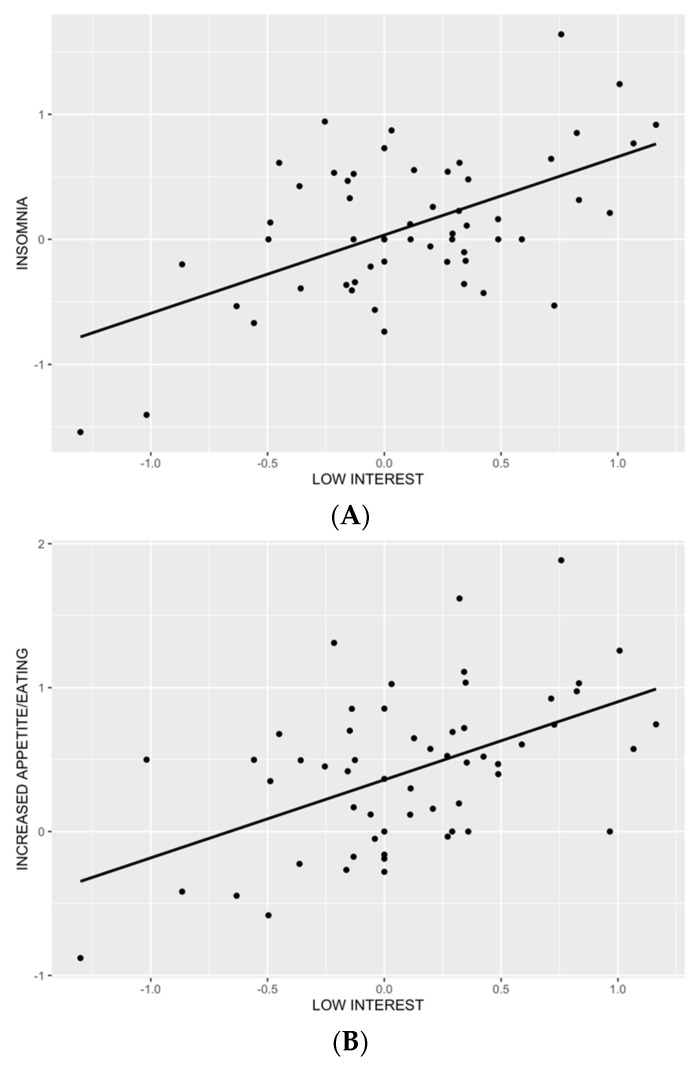
Scatterplot of a significant relationship between a premenstrual increase in low interest and insomnia (*p* = 0.0253; (**A**)), and a premenstrual increase in low interest and appetite/food intake (*p* = 0.0274; (**B**)).

The first linear regression model, explaining 24% of total variance, revealed that low interest (β = 0.48, *p* ≤ 0.05), but none of the other symptoms, was associated with insomnia (Table 2). Visual inspection of residual distribution suggested normality (Supplementary Figure S1A), which was confirmed by a non-significant Shapiro test (*p* = 0.40). The relationship between insomnia and low interest is depicted in Figure 1A.

None of the predictor variables (affective or psychological) were associated with hypersomnia from model 2 analysis (explaining 24% of total variance). Visual inspection of residual distribution suggested normality (Supplementary Figure S1B), which was confirmed by a non-significant Shapiro test (*p* = 0.08).

Results of the third model, explaining 22% of the total variance, showed a significant positive association between increased appetite/eating and low interest (β = 0.45, *p* ≤ 0.05), and none of the remaining affective and psychological premenstrual symptoms. Visual inspection of residual distribution suggested normality (Supplementary Figure S1C), which was confirmed by a non-significant Shapiro test (*p* = 0.71). The relationship between increased appetite/eating and low interest is depicted in Figure 1B. 

The fourth and last model in the evaluation of the relationships between behavioral and affective/psychological measures showed that an increase in premenstrual food cravings was marginally associated with difficulty concentrating (*p* = 0.056); this model explained 22% of the variance. Visual inspection of residual distribution suggested normality (Supplementary Figure S1D), which was confirmed by a non-significant Shapiro test (*p* = 0.33). 

Adjusted models matched the overall findings of unadjusted models, with the exception of the following three new associations: (1) in model 3, a negative correlation was identified between depressed mood and increased appetite/eating, (2) in model 4, a negative correlation was identified between depressed mood and food cravings, and (3) in model 4, a positive correlation was identified between difficulty concentrating and food cravings. These results are presented in Supplementary Table S4.

### 3.3. Relationships between Premenstrual Functionality and Behavioral Symptoms 

**Figure 2:**
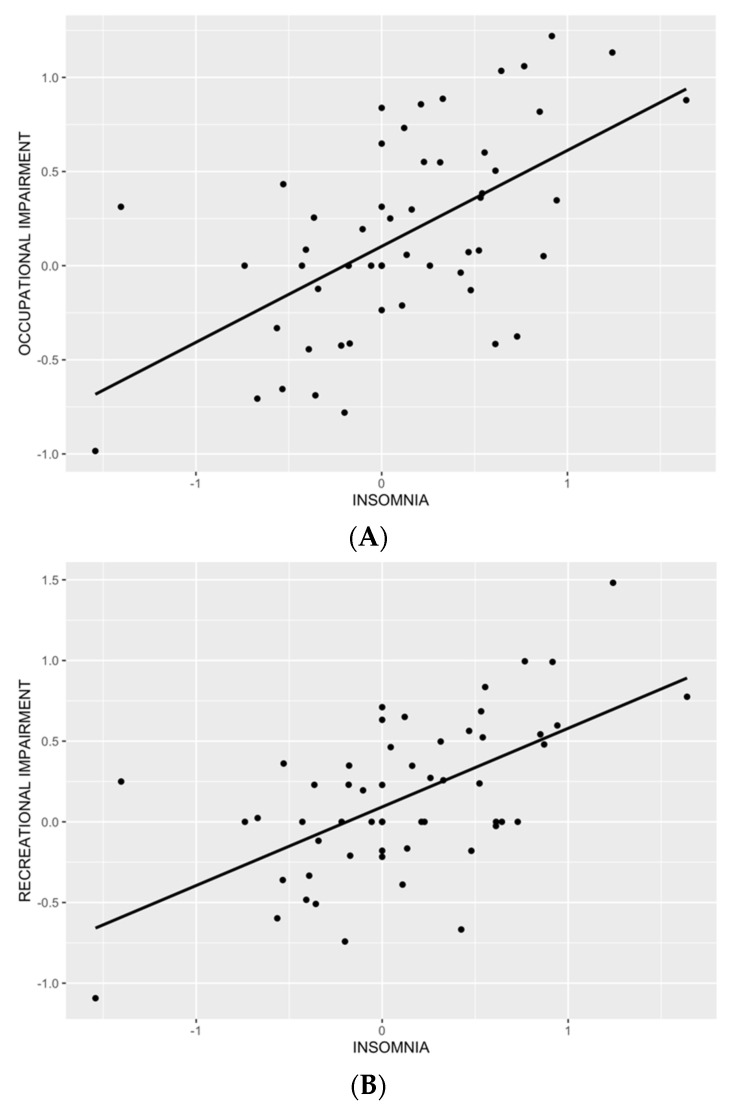

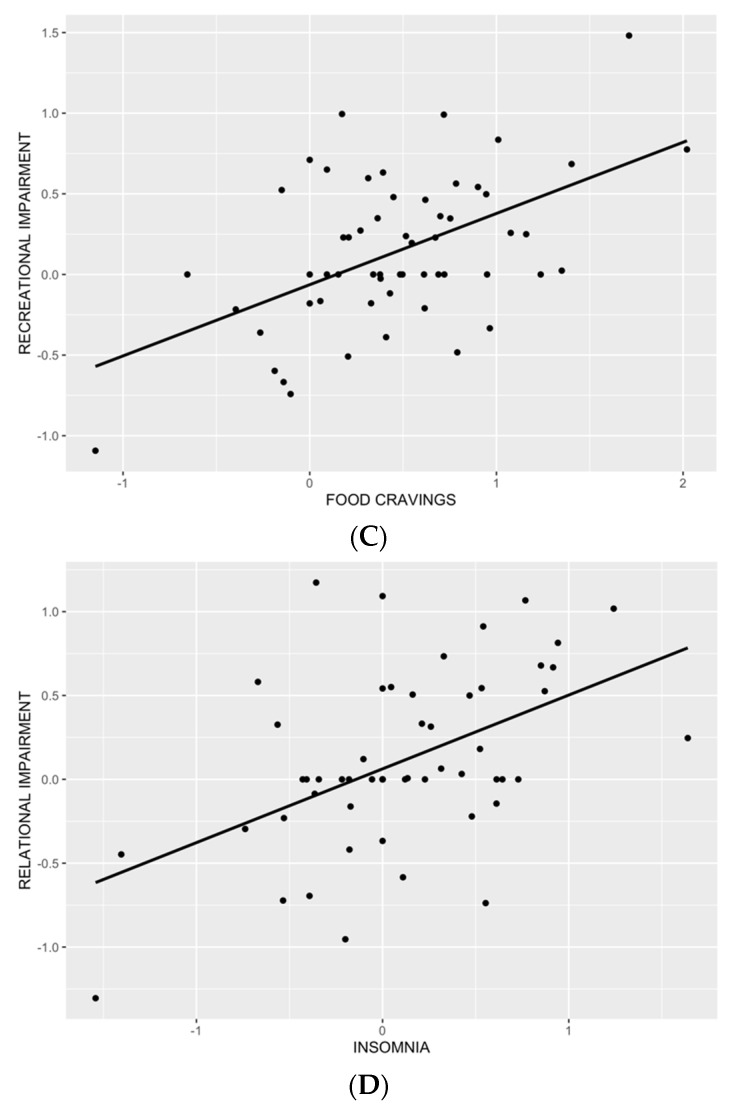
Scatterplot of a significant relationship between occupational impairment and insomnia (*p* = 0.00096; (**A**)), recreational impairment and insomnia (*p* = 0.000586; (**B**)), recreational impairment and food cravings (*p* = 0.020611; (**C**)), and relational impairment and insomnia (*p* = 0.00554; (**D**)).

The first model, examining the relationship between occupational impairment and behavioral symptoms, showed that insomnia (β = 0.39, *p* ≤ 0.001) and none of the remaining predictors were associated with occupational impairment (Table 3). Visual inspection of residual distribution suggested normality (Supplementary Figure S2A), which was confirmed by a non-significant Shapiro test (*p* = 0.18). The relationship between occupational impairment and insomnia is depicted in Figure 2A. 

The second model, examining the relationship between recreational impairment and behavioral symptoms, showed that insomnia (β = 0.35, *p* ≤ 0.001) and food cravings (β = 0.30, *p* ≤ 0.05) were associated with recreational impairment. None of the remaining predictors showed a significant association. Visual inspection of residual distribution suggested normality (Supplementary Figure S2B), which was confirmed by a non-significant Shapiro test (*p* = 0.50). The relationship between recreational impairment and insomnia is depicted in Figure 2B, and between recreational impairment and food cravings in Figure 2C.

The final model, explaining the relationship between relational impairment and behavioral symptoms, showed that insomnia (β = 0.35, *p* ≤ 0.01) and none of the remaining predictors showed a significant association. Visual inspection of residual distribution suggested normality (Supplementary Figure S2C), which was confirmed by a non-significant Shapiro test (*p* = 0.62). The relationship between relational impairment and insomnia is depicted in Figure 2D.

Associations between insomnia and occupational impairment, and insomnia and relational impairment remained significant in the adjusted models. The association between relational impairment and insomnia was marginally significant in the adjusted model. Finally, in the adjusted model, there was a positive association between hypersomnia and recreational impairment. These results are presented in Supplementary Table S5. 

## 4. Conclusions

In summary, the results of the present study highlight the importance of apathy in the expression of behavioral symptomatology of PMS/PMDD, as well as the importance of premenstrual insomnia on impairment experienced in the late luteal subphase of the menstrual cycle. Specifically, we identified significant positive associations between apathy and insomnia, and apathy and increased appetite/eating. Insomnia was significantly associated with all forms (occupational, recreational, and relational) of impairment in the premenstruum. 

A multifactorial process, loss of interest or apathy likely consists of several mechanisms—or combinations of mechanisms—which underlie its expression [34]. Fundamental to apathy are reduced voluntary, self-initiated, and goal-directed activities [35]. Our analysis evaluated the overall apathy; however, a future study may explore whether associations we uncovered here between apathy and both insomnia and overeating in the premenstruum are related to apathy operationalized according to the “Behavioral”, “Cognitive” or ‘Emotional’ subtype, which relate to different regions of the corticostriatal system [34]. 

Independent of the menstrual cycle phase, poor sleep quality and apathy, as well as apathy and overeating, are seemingly related. Disrupted sleep is linked to apathy across several neurologic conditions [36,37,38,39], and apathy seems to be related to weight loss success. Recruiting 101 apathetic obese individuals, Desouza et al. [40] showed that apathy improvement (resulting from treatment effects) was significantly associated with a successful weight loss. Our study is consistent with these findings, demonstrating that premenstrual apathy is linked to a premenstrual increase in appetite/eating. Furthermore, when we entered the total duration of endogenous hormone exposure in the adjusted model, depressed mood, in addition to apathy, was linked to increased appetite/eating. These results show that premenstrual eating is unrelated to anxiety, irritability, or anger, highlighting absence of connection with negative valence systems in the research domain criteria (RDoC). In the unadjusted model, the present analysis identified a strong association between insomnia and all three forms (occupational, recreational, and relational) impairment.

Summarizing research to date, Baker et al. [41] found a decrease in the duration of REM sleep episodes in the luteal phase of the menstrual cycle. Whether these REM sleep changes relate to objectively and subjectively measured changes in apathy in PMS/PMDD should be further explored. Moreover, in addition to the present findings, there is also evidence of a biological basis behind changes in sleep patterns. The increasing levels of progesterone during the early luteal subphase followed by a peak during the mid-luteal subphase were shown to be proportionate to sleep interruption [42]. The thermogenic property of progesterone leads to an increase in core body temperature, and this thermal response may be associated with the negative effects of progesterone on sleep [43,44].

The present finding highlights the critical need for the treatment of insomnia in a segment of reproductive-age women. Cognitive Behavioral Therapy for insomnia (CBT-I) is preferred as first-line therapy for chronic insomnia in most patients and is the standard of care [45]. Alternatively, two new medications became recently available for the treatment of insomnia. The Food and Drug Administration (FDA) approved suvorexant (Belsomra, Merck & Co., Rahway, NJ, 07065, USA) in 2014, and subsequently lemborexant (Dayvigo, Eisai Inc., Nutley, NJ, 07110, USA) in 2019; these two medications belong to a class of drugs called “dual orexin receptor antagonists” (DORA) and demonstrate a milder adverse event profile as well as absence of rebound or withdrawal effects compared to the older GABA-modulating medications for insomnia treatment [46]. Reproductive-age women suffering from insomnia in the premenstruum may benefit from CBT-I or a DORA treatment, which would, hypothetically, improve their functionality and quality of life.

Our study highlights the importance of considering behavioral symptomatology in the premenstruum. Insomnia [47] and disordered eating [48] in reproductive-age women have profound effects on cardiometabolic risk. Therefore, targeted treatment of these symptoms is of utmost importance. Our study is a longitudinal evaluation involving collection of self-reported data using a validated questionnaire designed to measure a broad range of premenstrual symptomatology. The self-reported nature of the survey, and its broad coverage of symptomatology, are study limitations. Therefore, these findings ideally should be replicated using objective and subjective methods of assessment, specifically designed to assess sleep, and eating behaviors. The prospective nature of symptom collection, as well as the adjustment for symptoms experienced in the follicular phase, are the study strengths. 

Identifying associations between premenstrual symptoms can improve the use of novel pharmacologic and non-pharmacologic therapeutics. The present study enhances our understanding of PMS/PMDD etiology as it identified co-expression of apathy and behavioral symptoms related to sleep and eating. Moreover, it highlighted the importance of sleep in the premenstruum on different forms of function. This can inform treatment approaches, thereby improving care for patients suffering from premenstrual symptomatology linked to chronic disease conditions. 

## Figures and Tables

**Table 1:** Demographic, Anthropomorphic and Psychological Characteristics of Study Participants.

Variable	Mean (SD)	Percent
Age	25.98 (4.69)	
Race		
White		33.33
Black or African American		15.78
American Indian/Alaska Native		1.75
Asian		36.84
Native Hawaiian or Other Pacific Islander		0
More than one race		5.26
Unknown/do not want to specify		7.01
Ethnicity		
Hispanic		15.78
Non-Hispanic		80.70
Do not know/Do not want to specify		3.50
Student Status		
Yes		47.36
No		52.63
Marital Status		
Single, never married		91.22
Married		8.77
Age of Menarche	12.05 (1.07)	
BMI	24.48 (4.37)	
Waist Circumference (cm)	79.63 (4.37)	

**Table 2:** Relationships between behavioral, affective and psychological premenstrual symptoms.

Symptom	Estimate	Standard Error	T Value	*p*-Value	Model Characteristics
Insomnia					
Depressed mood	−0.071033	0.202361	−0.351	0.7271	Residual standard error: 0.5061 on 49 degrees of freedom; Multiple R-squared: 0.327; Adjusted R-squared: 0.2309; F-statistic: 3.401 on 7 and 49 DF, *p*-value: 0.004865
Anxiety	0.006171	0.153829	0.04	0.9682	
Mood swings	−0.068889	0.19377	−0.356	0.7237	
Anger	0.096548	0.173253	0.557	0.5799	
Low interest	0.486635	0.210894	2.307	0.0253 *	
Difficulty concentrating	0.182399	0.189285	0.964	0.34	
Felt overwhelmed	0.040668	0.168132	0.242	0.8099	
Hypersomnia					
Depressed mood	0.19182	0.188628	1.017	0.314	Residual standard error: 0.4717 on 49 degrees of freedom; Multiple R-squared: 0.2437; Adjusted R-squared: 0.1356; F-statistic: 2.255 on 7 and 49 DF; *p*-value: 0.04517
Anxiety	0.09615	0.14339	0.671	0.506	
Mood swings	0.073938	0.18062	0.409	0.684	
Anger	−0.077098	0.161495	−0.477	0.635	
Low interest	0.103627	0.196582	0.527	0.6	
Difficulty concentrating	0.198554	0.176439	1.125	0.266	
Felt overwhelmed	0.008952	0.156722	0.057	0.955	
Increased appetite/eating					
Depressed mood	−0.2243	0.19172	−1.17	0.2477	Residual standard error: 0.4794 on 49 degrees of freedom; Multiple R-squared: 0.3138; Adjusted R-squared: 0.2157; F-statistic: 3.201 on 7 and 49 DF; *p*-value: 0.007163
Anxiety	0.07801	0.14574	0.535	0.5949	
Mood swings	0.09274	0.18358	0.505	0.6157	
Anger	0.15952	0.16414	0.972	0.3359	
Low interest	0.4543	0.1998	2.274	0.0274 *	
Difficulty concentrating	0.09768	0.17933	0.545	0.5884	
Felt overwhelmed	−0.08705	0.15929	−0.547	0.5872	
Food cravings					
Depressed mood	−0.095265	0.208594	−0.457	0.6499	Residual standard error: 0.5216 on 49 degrees of freedom; Multiple R-squared: 0.2241; Adjusted R-squared: 0.1133; F-statistic: 2.022 on 7 and 49 DF; *p*-value: 0.07104
Anxiety	−0.008728	0.158568	−0.055	0.9563	
Mood swings	0.12784	0.199739	0.64	0.5251	
Anger	0.210877	0.17859	1.181	0.2434	
Low interest	0.009962	0.21739	0.046	0.9636	
Difficulty concentrating	0.381662	0.195116	1.956	0.0562	
Felt overwhelmed	−0.194214	0.173312	−1.121	0.2679	

* *p* ≤ 0.05.

**Table 3:** Relationships between premenstrual functionality and behavioral symptoms.

Symptom	Estimate	Standard Error	T Value	*p*-Value	Model Characteristics
Occupational impairment					
Insomnia	0.39341	0.11237	3.501	0.00096 ***	Residual standard error: 0.4158 on 52 degrees of freedom Multiple R-squared: 0.3835, Adjusted R-squared: 0.336 F-statistic: 8.086 on 4 and 52 DF, *p*-value: 3.794 × 10^−5^
Hypersomnia	0.08241	0.1282	0.643	0.52317	
Food cravings	0.03363	0.14688	0.229	0.8198	
Increased appetite/eating	0.17491	0.16027	1.091	0.28015	
Recreational impairment					
Insomnia	0.35136	0.09594	3.662	0.000586 ***	Residual standard error: 0.355 on 52 degrees of freedom Multiple R-squared: 0.4755, Adjusted R-squared: 0.4351 F-statistic: 11.78 on 4 and 52 DF, *p*-value: 6.912 × 10^−7^
Hypersomnia	0.14466	0.10946	1.322	0.1921	
Food cravings	0.29946	0.1254	2.388	0.020611 *	
Increased appetite/eating	−0.05736	0.13684	−0.419	0.676798	
Relational impairment					
Insomnia	0.35352	0.12213	2.895	0.00554 **	Residual standard error: 0.355 on 52 degrees of freedom Multiple R-squared: 0.4755, Adjusted R-squared: 0.4351 F-statistic: 11.78 on 4 and 52 DF, *p*-value: 6.912 × 10^−7^
Hypersomnia	0.08699	0.13934	0.624	0.53518	
Food cravings	−0.08686	0.15963	−0.544	0.58868	
Increased appetite/eating	0.20062	0.17419	1.152	0.25468	

* *p* ≤ 0.05; ** *p* ≤ 0.01, *** *p* ≤ 0.001.

## Data Availability

The data presented in this study are available on request from the corresponding author.

## Supplementary Materials

The following supporting information can be downloaded at: https://www.mdpi.com/article/10.3390/brainsci12070814/s1, Table S1: Domain type and symptoms of PMDD as specified in DSM-5 and DRSP; Table S2: Means and standard deviations of individual symptoms in mid-follicular and late luteal subphases; Table S3: Demographic characteristics according to diagnosis; Table S4: Adjusted models evaluating premenstrual symptom relationships; Table S5: Adjusted models evaluating the relationship between behavioral symptoms and functionality. Figure S1: QQ plots of the four models evaluating relationships between behavioral and affective/physiological premenstrual symptoms; Figure S2: QQ plots of the four models evaluating relationships between functionality and behavioral symptoms.
