# Supplementary material for: Behavioral Symptomatology in the Premenstruum
Source: Brain Sci. 2022 Jun 22;12(7):814. doi: 10.3390/brainsci12070814 (PMC9312467; doi:10.3390/brainsci12070814)

Supplementary Table S1. Domain type and symptoms of PMDD as specified in DSM-5 and DRSP.

DSM-5 Symptoms	DRSP Question	Inclusion in Present Analysis
1. Marked affective lability (e.g., mood swings, feeling suddenly sad or tearful, or increased sensitivity to rejection*#	5. Had mood swings (e.g., suddenly felt sad or tearful)<br>DRSP 6. Was more sensitive to rejection or my feelings were easily hurt	Included as affective symptom
2. Marked irritability or anger or increased interpersonal conflicts*#	7. Felt angry, irritable<br>8. Had conflicts or problems with people	Included as affective symptom
3. Marked depressed mood, feelings of hopelessness, or self-deprecating thoughts*#	1. Felt depressed, sad, down, or blue<br>2. Felt hopeless<br>3. Felt worthless or guilty	Included as affective symptom
4. Marked anxiety, tension, and/or feelings of being keyed up or on edge*#	4. Felt anxious, tense, keyed up, or on edge	Included as affective symptom
5. Decreased interest in usual activities (e.g., work, school, friends, hobbies)	9. Had less interest in usual activities (e.g., work, school, friends, hobbies)	Included as psychological symptom
6. Subjective difficulty in concentration	10. Had difficulty concentrating	Included as psychological symptom
7. A sense of being overwhelmed or out of control#	16. Felt overwhelmed or that I could not cope<br>17. Felt out of control	Included as psychological symptom
8. Marked change in appetite; overeating; or specific food cravings	12. Had increased appetite or overate<br>13. Had cravings for specific foods	Included as behavioral symptom
9. Hypersomnia or insomnia	14. Slept more, took naps, found it hard to get up when intended<br>15. Had trouble getting to sleep or staying asleep	Included as behavioral symptom
10. Lethargy, easy fatigability, or marked lack of energy	11. Felt lethargic, tired, fatigued, or had a lack of energy	Not included – physical symptom
11. One physical symptom (for example, breast tenderness)	18. Had breast tenderness<br>19. Had breast swelling, felt bloated, or had weight gain<br>20. Had headache<br>21. Had joint or muscle pain	Not included – physical symptom
* At least one symptom from items 1 to 4 in column 1 (DSM-5 Symptoms) be present for PMDD diagnosis<br># The first symptom inside the parentheses was included in the analysis		

Supplementary Table S2. Means and standard deviations of individual symptom effect sizes.

Premenstrual Symptom	Mean	Standard Deviation
DEPRESSION	0.0743	0.4758
MOOD SWINGS	0.2145	0.5203
ANGER	0.2030	0.5415
ANXIETY	0.1448	0.5740
DIFFICULTY CONCENTRATING	0.1262	0.5456
FELT OVERWHELMED	0.1646	0.5190
LOW INTEREST	0.1096	0.5070
INCREASED APPETITE	0.4188	0.5414
FOOD CRAVINGS	0.4671	0.5540
HYPERSOMNIA	0.1031	0.5074
INSOMNIA	0.1032	0.5770
OCCUPATIONAL IMPAIRMENT	0.1552	0.5102
RECREATIONAL IMPAIRMENT	0.1427	0.4723
RELATIONAL IMPAIRMENT	0.1081	0.5127

Supplementary Table S3. Demographic characteristics according to diagnosis.

Demographic Variable	Category	Diagnosis			
		PMDD(n=8)	PMS(n=25)	Healthy(n=24)	p value
Race	White	3 (37.5)	7 (28.0)	9 (37.5)	0.8499
	Black or African American	2 (25.0)	3 (12.0)	4 (16.7)	
	American Indian/Alaska Native	0 (0.0)	0 (0.0)	1 (4.2)	
	Asian	2 (25.0)	12 (48.0)	7 (29.2)	
	More than one race	1 (12.5)	1 (4.0)	1 (4.2)	
	Unknown/do not want to specify	0 (0.0)	2 (8.0)	2 (8.3)	
Age		24.50 (4.04)	25.48 (5.02)	27.00 (4.52)	0.338
BMI		25.15 (4.35)	24.92 (5.18)	23.82 (3.60)	0.648
Ethnicity	Hispanic	1 (12.5)	3 (12.0)	5 (20.8)	0.4187
	Non-Hispanic	6 (75.0)	22 (88.0)	18 (75.0)	
	Unknown/do not want to specify	1 (12.5)	0 (0.0)	1 (4.2)	
Student Status	Yes	4 (50.0)	12 (48.0)	11 (45.8)	0.976
	No	4 (50.0)	13 (52.0)	13 (54.2)	
Marital Status	Single	8 (100.0)	22 (88.0)	22 (91.7)	0.8418
	Married	0 (0.0)	3 (12.0)	2 (8.3)	
Income	Less than $20,000	5 (62.5)	16 (64.0)	8 (33.3)	0.058
	$20,000-$34,999	1 (12.5)	0 (0.0)	6 (25.0)	
	$35,000-$49,999	1 (12.5)	5 (20.0)	3 (12.5)	
	$50,000-$74,999	1 (12.5)	2 (8.0)	7 (29.2)	
	$75,000 or more	0 (0.0)	2 (8.0)	0 (0.0)	
Age of Menarche		12.40 (1.14)	12.12 (0.93)	11.83 (1.27)	0.594

Supplementary Table S4. Adjusted models evaluating premenstrual symptom relationships.

SYMPTOM	ESTIMATE	STANDARD ERROR	T VALUE	P VALUE
INSOMNIA				
DEPRESSION	-0.39557	0.294106	-1.345	0.1912
ANXIETY	0.076034	0.196937	0.386	0.7028
MOOD SWINGS	0.105953	0.282468	0.375	0.7109
ANGER	0.277414	0.243891	1.137	0.2666
LOW INTEREST	0.599109	0.262505	2.282	0.0316*
DIFFICULTY CONCENTRATING	-0.043421	0.25985	-0.167	0.8687
FELT OVERWHELMED	0.281331	0.236662	1.189	0.2462
AGE	0.019214	0.022362	0.859	0.3987
AGE OF MENARCHE	-0.005492	0.100084	-0.055	0.9567
HYPERSOMNIA				
DEPRESSION	0.168902	0.266552	0.634	0.532
ANXIETY	0.123401	0.178487	0.691	0.496
MOOD SWINGS	-0.1103	0.256004	-0.431	0.67
ANGER	-0.136986	0.221042	-0.62	0.541
LOW INTEREST	0.236378	0.237912	0.994	0.33
DIFFICULTY CONCENTRATING	0.327429	0.235506	1.39	0.177
FELT OVERWHELMED	0.037541	0.21449	0.175	0.863
AGE	0.001869	0.020267	0.092	0.927
AGE OF MENARCHE	0.042707	0.090708	0.471	0.642
INCREASED APPETITE/EATING				
DEPRESSION	-0.58194	0.242341	-2.401	0.0244*
ANXIETY	0.163369	0.162275	1.007	0.3241
MOOD SWINGS	0.252509	0.232752	1.085	0.2888
ANGER	0.080785	0.200965	0.402	0.6913
LOW INTEREST	0.466179	0.216302	2.155	0.0414*
DIFFICULTY CONCENTRATING	0.296309	0.214115	1.384	0.1791
FELT OVERWHELMED	-0.065601	0.195008	-0.336	0.7395
AGE	-0.021103	0.018426	-1.145	0.2634
AGE OF MENARCHE	0.006783	0.082469	0.082	0.9351
FOOD CRAVINGS				
DEPRESSION	-0.575608	0.24692	-2.331	0.0285*
ANXIETY	0.088026	0.16534	0.532	0.5993
MOOD SWINGS	0.423518	0.237149	1.786	0.0868
ANGER	0.191678	0.204761	0.936	0.3585
LOW INTEREST	-0.013931	0.220389	-0.063	0.9501
DIFFICULTY CONCENTRATING	0.488043	0.21816	2.237	0.0348*
FELT OVERWHELMED	-0.036474	0.198692	-0.184	0.8559
AGE	-0.002857	0.018775	-0.152	0.8803
AGE OF MENARCHE	-0.095875	0.084027	-1.141	0.2651

Supplementary Table S5. Adjusted models evaluating relationship between behavioral symptoms and functionality.

SYMPTOM	ESTIMATE	STANDARD ERROR	T VALUE	P VALUE
OCCUPATIONAL IMPAIRMENT				
INSOMNIA	0.3122	0.14097	2.215	0.0354 *
HYPERSOMNIA	0.10048	0.16644	0.604	0.5511
FOOD CRAVINGS	0.25927	0.18767	1.381	0.1785
INCREASED APPETITE	0.06005	0.19803	0.303	0.7641
AGE	0.01244	0.01583	0.786	0.4388
AGE OF MENARCHE	-0.0166	0.07087	-0.234	0.8166
RECREATIONAL IMPAIRMENT				
INSOMNIA	0.26824	0.11119	2.412	0.0229 *
HYPERSOMNIA	0.286	0.13128	2.178	0.0383 *
FOOD CRAVINGS	0.35444	0.14803	2.394	0.0239 *
INCREASED APPETITE	-0.12805	0.1562	-0.82	0.4195
AGE	-0.0141	0.01249	-1.129	0.2689
AGE OF MENARCHE	-0.05205	0.0559	-0.931	0.3601
RELATIONAL IMPAIRMENT				
INSOMNIA	0.281846	0.161617	1.744	0.0926 .
HYPERSOMNIA	0.009164	0.190822	0.048	0.962
FOOD CRAVINGS	0.076698	0.215162	0.356	0.7243
INCREASED APPETITE	0.189924	0.22704	0.837	0.4102
AGE	0.018914	0.018151	1.042	0.3066
AGE OF MENARCHE	0.006445	0.081253	0.079	0.9374

Supplementary Figure S1. QQ plots of the four models evaluating relationships between behavioral and affective/physiological premenstrual symptoms.

1A. INSOMNIA

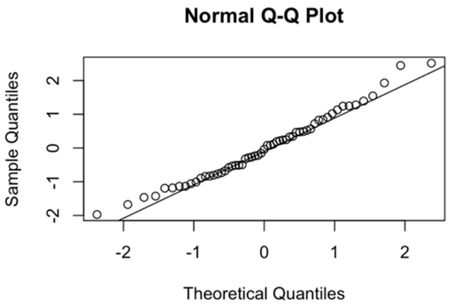

1B. HYPERSOMNIA

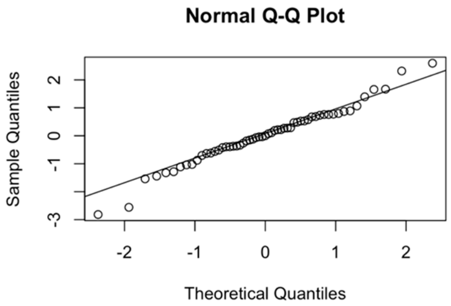

1C. INCREASED APPETITE/EATING

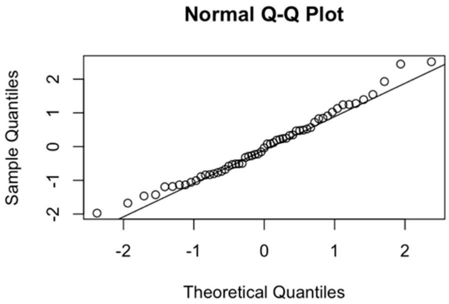

1D. FOOD CRAVINGS

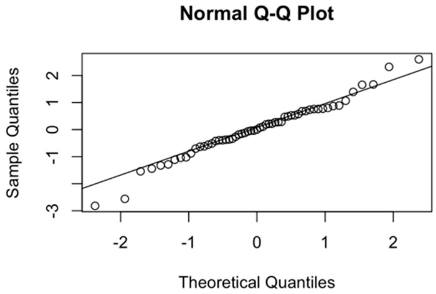

Supplementary Figure S2. QQ plots of the four models evaluating relationships between functionality and behavioral symptoms.

2A. OCCUPATIONAL IMPAIRMENT

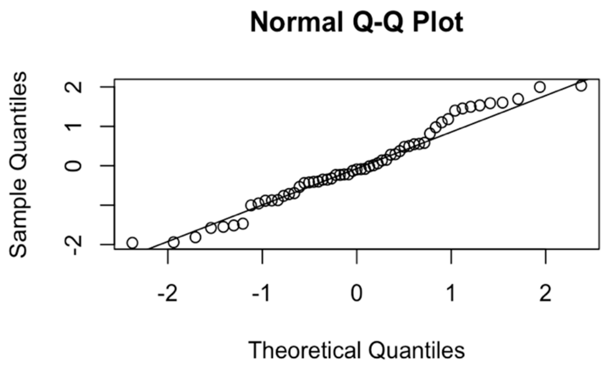

2B. RECREATIONAL IMPAIRMENT

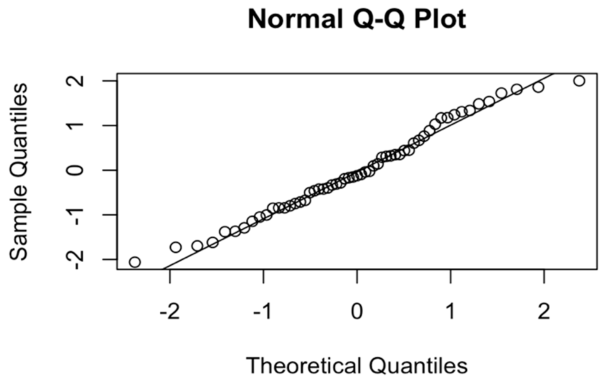

2C. RELATIONAL IMPAIRMENT

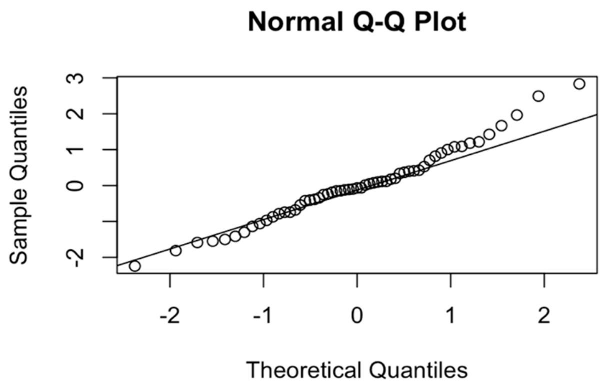

Supplement: Supplementary file 1 [file brainsci-12-00814-s001.zip › brainsci-1703813-SI.pdf]
